# Single-Cell Multi-Omics Reveal Gene Regulatory Mechanisms Underlying Cardiac Embryonic Development

**DOI:** 10.3390/genes17040414

**Published:** 2026-03-31

**Authors:** Enqi Feng, Xuejia Zheng, Feng Zhu, Liu Xiang, Chengcheng Liu, Leping Wang, Yanni Cao, Yong Dai

**Affiliations:** 1 School of Mathematics and Big Data, Anhui University of Science & Technology, Huainan 232001, China; 15397844554@163.com (E.F.); i61914027@outlook.com (C.L.); 17330699973@163.com (L.W.); 2 The First Hospital, Anhui University of Science & Technology, Huainan 232001, China; z1967126157@gmail.com (X.Z.); zhudc6187@gmail.com (F.Z.); 3 School of Computer Science and Engineering, Anhui University of Science & Technology, Huainan 232001, China; xl17634447214@163.com; 4 School of Artificial Intelligence, Anhui University of Science & Technology, Huainan 232001, China; 5 School of Medicine, Anhui University of Science & Technology, Huainan 232001, China; 6 Anhui Provincial Academician Workstation of Anhui University of Science & Technology for Autoimmune Disease Diagnostic Technology, Huainan 232001, China

**Keywords:** embryonic heart development, developmental genetics, congenital heart disease (CHD), single-cell multi-omics, single-cell RNA sequencing (scRNA-seq), single-cell assay for transposase-accessible chromatin sequencing (scATAC-seq), epigenetic regulation

## Abstract

**Background/Objectives:** Cardiac embryonic development is a highly coordinated and dynamic process governed by precise spatiotemporal gene regulation. Increasing evidence indicates that cellular heterogeneity and lineage specification during heart development are tightly controlled by complex gene regulatory networks (GRNs) and epigenetic mechanisms. Recent advances in single-cell multi-omics technologies provide unprecedented resolution to dissect these regulatory processes. This review aims to summarise current applications of single-cell multi-omics approaches to elucidate gene regulatory mechanisms underlying cardiac embryogenesis and their implications for congenital heart disease (CHD). **Methods:** We systematically reviewed recent literature on single-cell RNA sequencing (scRNA-seq), single-cell assay for transposase-accessible chromatin sequencing (scATAC-seq), spatial transcriptomics, and integrative multi-omics analyses applied to embryonic heart development. Studies were analysed to evaluate how these technologies contribute to cell-type identification, lineage trajectory reconstruction, GRN inference, and epigenetic landscape characterisation. **Results:** Single-cell multi-omics approaches have enabled the construction of high-resolution cardiac cell atlases, revealing previously unrecognised cellular heterogeneity and transitional states during heart development. Integrative analyses of transcriptomic and chromatin accessibility data have provided insights into lineage commitment, key transcription factors, enhancer–promoter interactions, and dynamic GRNs. These findings have advanced understanding of developmental genetics in cardiac morphogenesis and offered new perspectives on the molecular mechanisms underlying CHD. **Conclusions:** Single-cell multi-omics technologies provide a powerful framework for investigating gene regulatory mechanisms during cardiac embryogenesis. Continued methodological refinement and integrative analyses are expected to further clarify developmental processes and facilitate translational insights into CHD.

## 1. Introduction

The heart is the first organ to form and function during embryonic development, with complex morphogenetic processes involving precise cell-fate decisions, lineage differentiation, and spatial organisation [1]. From mesoderm specialisation at the gastrula stage, through heart tube formation, cardiac looping, and chamber partitioning, to the final maturation of the outflow tract and septum, each stage depends upon the temporal and spatial coordination of multiple signalling pathways and the precise execution of gene regulatory networks (GRNs). The development of the embryonic heart is a sophisticated cascade process that is temporally and spatially regulated by multiple signalling pathways and transcription factors [2]. Any minor imbalance at key nodes during this process can disrupt the developmental cascade, leading to severe structural malformations of the heart [3,4].

Given the intricate nature of cardiac development, it is understandable that deviations from these precisely regulated processes can lead to congenital heart disease (CHD). CHD, representing one of the most prevalent birth defects, is often rooted in the impairment of discrete subsets of cardiac precursor cells; however, the precise transcriptional alterations within these precursors that culminate in organ-level structural anomalies remain largely uncharacterized [5].

**Figure 1:**
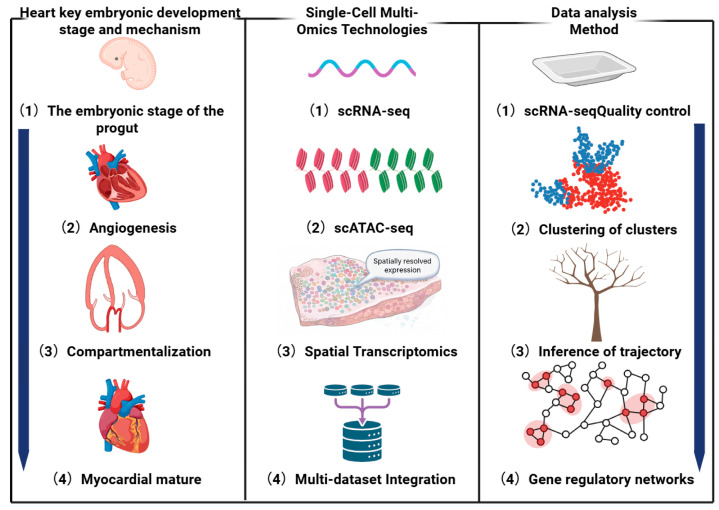
Overview of single-cell multi-omics technologies and data analysis in cardiac embryonic development.

In the past decade, the emergence of single-cell sequencing technologies has revolutionised the way we study heart development [6]. Single-cell RNA sequencing (scRNA-seq) can capture gene expression heterogeneity at the single-cell level, revealing cell type diversity and dynamic differentiation processes [7,8,9]. Single-cell assay for transposase-accessible chromatin sequencing (scATAC-seq) can analyse the changes in chromatin accessibility and reveal the epigenetic regulatory mechanism [10]. Technologies such as Cellular indexing of transcriptomes and epitopes by sequencing (CITE-seq) can simultaneously obtain transcriptome and protein expression information [11]. Spatial transcriptomics preserves the spatial information of cells in situ and helps to analyse tissue structures and cell interactions [12,13]. The application of these technologies has generated vast and complex datasets, providing unprecedented opportunities for constructing cellular maps of heart development, analysing cell differentiation paths, and inferring GRNs. This review will systematically summarise the application of single-cell multi-omics technology in cardiac embryonic development, focusing on how it deciphers cellular heterogeneity, dynamic trajectories, and molecular regulatory networks. To provide a clearer research perspective, we establish a logical framework for technology selection: prioritising specific omics modalities based on the biological requirements of each developmental stage—for instance, utilising scATAC-seq to identify upstream drivers during gastrulation, while employing CITE-seq to characterise downstream functional effects during cardiomyocyte maturation. As shown in Figure 1, the review will successively explore the single-cell multi-omics technology system, its specific applications at various stages of cardiac development, the data analysis methods supporting its applications, and prospects.

## 2. Development of Single-Cell Sequencing Technology and Its Application in Cardiac Embryo Development

The iterative development of single-cell sequencing technology has provided high-resolution tools for the analysis of the complex process of cardiac embryonic development. The technological evolution from transcriptome, epigenetic inheritance, to spatial multi-omics continues to expand our understanding of the heterogeneity, dynamic regulation, and microenvironment interaction of cardiac development cells [14,15]. The core advantage of scRNA-seq is its ability to resolve the cellular heterogeneity of tissues to create a high-resolution cellular map and gene expression panorama for development [14].

### 2.1. Single-Cell RNA Sequencing in Cardiac Embryonic Development

scRNA-seq technology, by capturing transcriptome information of individual cells, breaks the limitation of averaging in traditional bulk sequencing and becomes a core means for analysing the heterogeneity of cardiac developmental cells [16]. Early scRNA-seq platforms, including Switching mechanism at the 5′ end of the RNA template (Smart)-seq2, could obtain full-length transcripts, but their throughput was relatively low [17,18]. Droplet-based platforms (such as 10x Genomics Chromium) have achieved high-throughput single-cell capture, laying the foundation for the construction of large-scale cardiac developmental cell maps [19,20].

Cardiac embryonic development is a continuous dynamic process covering embryogenesis to the neonatal stage. scRNA-seq has shown strong application value in several core research directions in this field by virtue of its high-resolution advantage. Firstly, the analysis of fine cell subpopulations is the foundation of the research. Feng et al. systematically identified core cell types, such as myocardial, endocardial, and epicardial progenitors, through single-cell transcriptome analysis of mouse and human embryonic hearts. Furthermore, this study characterized the heterogeneous subpopulations within the second heart field (SHF), with a focus on Iroquois homeobox 1 (IRX1)-positive progenitor cells. It has clarified its key role in the formation of the outflow tract and the right ventricle, providing precise cellular targets for subsequent functional studies [5]. Secondly, dynamic tracking of developmental trajectories is an extension of cell typing. By leveraging methods such as quasi-temporal analysis and cell cycle analysis, researchers have successfully mapped the developmental trajectory of human pluripotent stem cell-derived cardiomyocytes (hPSC-CMs) in four engineered cardiac tissue models, such as the maturation process of hPSC-CMs in human ventricular cardiac anisotropic sheets (hvCAS) and human ventricular cardiac organoid chambers (hvCOC). It revealed the maturation patterns and key regulatory genes of cardiomyocytes and other cardiac cells in the engineered tissue environment, and this result was verified by different hPSC sources and repeated experiments, with high reliability [21]. Finally, the analysis of cell communication networks is a deep exploration of developmental regulatory mechanisms. By using tools such as CellChat and integrating data integration algorithms from single-cell and spatial transcriptomics, the interaction relationships between cardiomyocytes, endothelial cells, and fibroblasts (such as the regulatory role of the Notch signalling pathway in atrioventricular duct development) have been analysed. It also systematically surveyed the organisational relationships of various cell lineages in the developing heart [22], providing key clues for clarifying the cooperative regulatory network of heart development.

### 2.2. Cardiac Epigenetic Regulation via Single-Cell Chromatin Accessibility

Single-cell epigenomic sequencing technologies—including scATAC-seq, single-cell bisulfite sequencing (scBS-seq), and single-cell Chromatin Immunoprecipitation sequencing (scChIP-seq)—elucidate the epigenetic regulatory network governing cardiac development from multiple dimensions, including chromatin conformation, DNA modifications, and histone modifications [23]. Among them, the high-resolution analysis of chromatin accessibility by scATAC-seq is the core link connecting various epigenetic events [24]. The principle is to utilize transposase accessible chromatin sequencing technology to locate the open regions of chromatin at the single-cell level and directly identify the DNA sites that can bind to regulatory proteins such as transcription factors [25]. Chromatin accessibility, as the fundamental level of epigenetic regulation, not only directly determines the binding efficiency of transcription factors but also participates in the construction of cell-specific epigenetic states by regulating the targeted binding of methylases and histone modification enzymes to DNA, providing a key entry point for analysing the GRN of heart development [26,27].

The epigenetic regulatory mechanism of heart development is gradually being clarified through the combined application of single-cell epigenetic sequencing [24]. The integrated analysis of scATAC-seq and scChIP-seq confirmed that the transcription factor ISL1 exerts pioneer factor activity in cardiac progenitor cells—it can bind to closed chromatin regions, recruit Switch/Sucrose Non-Fermentable (SWI/SNF) chromatin–remodeling complexes open chromatin conformations [10], simultaneously promote the enrichment of active modifications such as histone acetylation, and synergistically activate myocardial differentiation-related enhancers to shape the epigenetic blueprint for cell fate selection [28]. Further research shows that the dynamic changes in chromatin accessibility are a prerequisite for the subsequent establishment of DNA methylation modifications: The combined analysis of scATAC-seq and scBS-seq revealed that key enhancers for cardiac development, including the downstream enhancer of the T-Box transcription factor 5 (TBX5) gene, would first “pre-position” into an open state through increased chromatin accessibility during early development, thereby guiding precise modifications such as DNA demethylation [5]. The loss of chromatin access in the downstream enhancer region of the TBX5 gene promotes abnormal methylation deposition. This synergistic anomaly is closely related to CHDs such as atrial septal defect and ventricular septal defect and inhibits the normal expression of TBX5 [29,30]. The demethylation treatment mediated by 5-aza-2-deoxycytidine can simultaneously restore the chromatin accessibility and TBX5 expression level in this region, thereby improving the differentiation defects of cardiomyocytes [31]. Although the techniques of scBS-seq and scChIP-seq are relatively difficult, the combined application with scATAC-seq has become a core strategy for analyzing the epigenetic hierarchical regulation of cardiac development [30].

### 2.3. Spatial Transcriptomics to Reveal Spatial Localization and Signaling

The emergence of spatial transcriptomics technology has effectively supplemented the missing spatial dimensions of scRNA-seq. Through algorithms such as SEU-TCA [32], Lin et al. have been able to precisely locate cardiac progenitor cells and analyze the signal interactions in their spatial microenvironment [33]. Studies have revealed that organ primordial-determining regions in the early mouse embryo synergistically integrate WNT inhibitory and BMP activating signals to drive the extracellular matrix (ECM) development of the heart and foregut, a process that is fundamentally linked to the early patterning of the ventral body wall [34].

High-resolution spatial transcriptomics techniques, such as multiplexed error-robust fluorescence in situ hybridization (MERFISH) technology, have enabled spatial gene expression analysis at single-cell resolution [35]. In a study of the human embryonic heart, Farah et al. systematically analysed the spatial distribution and interaction patterns of 75 cell subsets using MERFISH [6]. They observed that endomucin-positive endothelial cells are in close spatial proximity to MYH6-positive cardiomyocytes, providing direct evidence for spatially specific activation of vascular endothelial growth factor (VEGF) signaling during angiogenesis. From a clinical perspective, such precise spatial mapping is crucial for pediatric cardiologists to understand the pathogenesis of CHDs. For instance, disruptions in these spatially coordinated endothelial–cardiomyocyte interactions or VEGF signalling gradients can lead to impaired trabeculation and ventricular non-compaction, offering a molecular explanation for structural anomalies frequently encountered in clinical practice [36].

### 2.4. Multi-Omics Integration in Cardiac Development Regulation

In the study of cardiac development, due to its own limitations, single-omics technology is difficult to comprehensively analyse the complex regulatory network of cardiac development. Therefore, multi-omics integration has become an inevitable trend. The integrated analysis of scRNA-seq and scATAC-seq can directly correlate the chromatin open state with the gene transcriptional activity, thereby analysing the enhancer–promoter regulatory network [37]. Through this method, Zhao et al. revealed that the chromatin opening dynamics of the GATA4 binding site were highly consistent with the expression timing of downstream genes in the process of cardiac development [38]. By integrating transcriptomic and proteomic information, CITE-seq enables high-resolution discrimination of cell types in heart development [39]. This technique functionally correlates the mRNA and protein expression of Myl4 and Tnni1 in monocytic diploid cardiomyocytes, which not only clarifies their key phenotypic characteristics but also provides direct evidence for their roles in cardiac repair, revealing the mechanism of cellular action in cardiac development and repair, and providing more abundant and accurate information for the study of cardiac development [40].

To make a clear comparison between these techniques, their application in cardiac embryo development is summarised in Table 1. Beyond individual strengths, the synergy of these tools follows a biological thread: scATAC-seq is prioritised for identifying upstream ‘pioneer’ drivers, scRNA-seq for defining cell-state transitions, and spatial transcriptomics for resolving localised signalling interactions that govern morphological changes.

## 3. Application of Single-Cell Multi-Omics in Various Stages of Cardiac Embryonic Development

Single-cell multi-omics technology has been widely used at all key stages of cardiac embryonic development, providing unprecedented insights from cell map construction to lineage tracing to regulatory mechanism resolution.

### 3.1. Gastrulation and Mesoderm Specification of Cardiac Progenitor Origin

At the earliest stage of heart development, the central biological question is how cell fate is initiated, and identifying the upstream regulators of this process remains a major challenge. scATAC-seq is particularly useful for revealing how chromatin accessibility primes the genome before overt gene expression changes occur, whereas scRNA-seq complements this by capturing the downstream transcriptional programs associated with mesoderm specification [41]. Using single-cell sequencing of E7.5-E7.75 mouse embryos, Zhao et al. showed that mesodermal cells segregate into Flk1^+^ cardiovascular lineages and T^+^ paraxial/segmental lineages [42]. Studies have shown that the transcription factor Mesp1 plays a key molecular switch role, ensuring the establishment and maintenance of cardiovascular progenitor cell identity by inhibiting the somite-determining factor T/Brachyury. Its conditional absence can lead to the abnormal formation of the embryonic germ layer in E7.5 mouse embryos [43]. Advanced transcriptomic analyses have further elucidated the specificity of signaling regulation; for instance, Mesp1 has been shown to activate key mesendoderm modulators, including Wnt3, Wnt5a, and Wnt5b [44]. Additionally, the Wnt inhibitor Dkk1 plays a pivotal role in inducing the development of chordal and prechordal mesoderm, as well as promoting cardiogenesis from non-cardiogenic mesodermal populations [44,45].

### 3.2. PDZ and Heart Tube Formation: A Stage Dominated by Spatial Signalling Coordination

During heart tube formation, the research priority shifts toward understanding how morphological changes are coordinated in three-dimensional space. For biological questions regarding how localised gradients (e.g., WNT and BMP) guide this morphogenesis, spatial transcriptomics is the superior modality compared to traditional dissociated cell sequencing. Spatial transcriptomic studies have shown that the organ primordium-determining region (PDZ) of mouse E7.75 embryos coordinates the synchronous development of heart and foregut primordia by constructing a signalling microenvironment that simultaneously expresses the WNT repressor Dkk1 and the BMP activator Bmp4 [34]. This synergistic signalling hub is also critical for the proper specification of the ventral body wall; from a clinical perspective, its disruption can lead to severe midline anomalies such as Pentalogy of Cantrell, which involves complex ventral wall defects and malformations of the heart [46]. Complementing spatial transcriptomic evidence of regionalised morphogen signalling, the Noseda group further demonstrated through single-cell transcriptional profiling and clonal analysis that PDGFRα-defined cardiogenic progenitor subpopulations exhibit distinct molecular programmes associated with lineage commitment, highlighting the importance of coordinated intercellular regulatory networks in shaping early cardiac morphogenesis [47]. At the stage of heart tube formation, scRNA-seq revealed that cardiomyocytes began to express contractile proteins (such as Tnnt2 and Myh6) and ion channels (such as Scn5a), and initiated the early interaction between endocardial cells and cardiomyocytes [48], laying the foundation for subsequent endocardial pad formation and cardiac ventricularization, which has a profound impact on the normal development of the heart.

### 3.3. SHF Progenitor Differentiation and Cardiac Chamberization

SHF progenitors are critical cell sources for the formation of the outflow tract and right ventricle. Feng et al. identified heterogeneous subsets of SHF progenitors, including IRX1-positive progenitors, through single-cell transcriptomic analysis of mouse and human embryonic hearts [34]. When combined with time-series analysis, scRNA-seq can further trace the divergent differentiation trajectories of SHF progenitor cells. Functional validation showed that IRX1 is a key regulator of SHF development, and its knockdown resulted in outflow tract and ventricular septal defects, underscoring the importance of proper migration and differentiation of IRX1^+^ progenitor cells. In addition, the development of SHF progenitors is synergistically regulated by BMP and FGF signalling [49,50]. During chamber formation, scRNA-seq revealed distinct roles of Notch signalling in different cardiac regions. In the atrioventricular canal, Notch signalling promotes mesenchymal transition and endocardial cushion formation, whereas in the ventricle, it regulates trabecular formation and cellular proliferation [51,52].

### 3.4. Cardiomyocyte Maturation and Metabolic Reprogramming

Cardiomyocyte maturation is the final critical stage of cardiac development, and the establishment of its function and phenotypic stability directly affects the normal physiological function of the heart [53]. As cardiac development progresses into the maturation stage, the scientific objective transitions from defining cell identity to evaluating downstream functional effectors. Technologies such as scRNA-seq and CITE-seq provide powerful tools for analysing this process [54]. Specifically, CITE-seq is prioritised here as it directly links transcriptomic data with protein-level functional markers, offering a more accurate assessment of physiological maturity. For instance, through this type of technology, studies have found that Myl4^+^Tnni1^+^ mononuclear diploid cardiomyocytes (MNDCM) persist from embryonic to adult hearts and may play an important role in the repair process after cardiac injury, providing a new perspective for understanding the functional plasticity of cardiomyocytes after maturation [55]. Multi-omics integration analysis further revealed that metabolic reprogramming is one of the core characteristics of cardiomyocyte maturation—the cellular energy metabolism pattern gradually shifts from relying on glycolysis in the embryonic stage to being dominated by oxidative phosphorylation in the mature stage [56]. Studies on hPSC-derived cardiomyocytes in three-dimensional (3D) organoids have confirmed that overexpression of peroxisome proliferator-activated receptor alpha (PPARα) can accelerate this metabolic conversion process, thereby promoting cardiomyocyte maturation [57]. In addition to metabolic reprogramming, RNA splicing regulation also contributes to the temporal coordination of cardiomyocyte maturation. RNA-binding proteins such as nuclear cap binding protein subunit 2 (NCBP2) regulate this process through selective splicing, thereby ensuring precise expression of functional genes and orderly activation of the associated regulatory network [40].

### 3.5. Multi-Omics Integration of Molecular Regulatory Networks

Multi-omics integration has greatly deepened our systematic understanding of the molecular regulatory networks underlying cardiac embryonic development by linking data from different molecular layers, such as the transcriptome and epigenome. Among these approaches, the combined application of scRNA-seq and scATAC-seq has become a core strategy because it directly links chromatin accessibility with transcriptional activity and enables analysis of enhancer–promoter regulatory relationships [58]. Ameen et al. discovered through this integration method that the chromatin open dynamics at the binding site of the key transcription factor GATA4 for cardiac development are highly consistent with the expression timing of its downstream target genes [37]. As a pioneer factor in cardiac progenitor cells, ISL1 can bind to closed chromatin regions and recruit SWI/SNF chromatin–remodeling complexes, thereby synergistically activating enhancers associated with myocardial differentiation and laying an epigenetic foundation for cell fate selection [10]. In addition, the analysis of regulatory mechanisms such as DNA methylation and long non-coding RNA (lncRNA) has further enriched the dimensions of the regulatory network. For instance, abnormal DNA methylation in TBX5-related regulatory regions is closely associated with CHD, including tetralogy of Fallot. lncRNA Braveheart regulates myocardial lineage determination through interaction with the polycomb repressive complex 2 (PRC2) complex. These findings jointly construct a multi-level cardiac development regulatory system [59,60].

The regulation of each key stage of cardiac embryonic development does not occur in isolation but instead emerges through the dynamic interplay of molecular networks across developmental stages. Integrating multi-omics technologies with functional experiments and animal models not only reveals how transcription factors, epigenetic modifications, and non-coding RNAs interact, but also clarifies how disruption of these regulatory networks contributes to the pathogenesis of CHD [2]. This systems-level understanding provides an important foundation for elucidating the developmental logic of the heart and for advancing the etiological analysis and potential treatment of CHD. At the same time, extracting these biological insights from increasingly complex datasets depends heavily on robust computational pipelines and statistical models, which are discussed in the following section.

## 4. Application of Data Analysis Methods and Statistical Models in Cardiac Development

**Figure 2:**
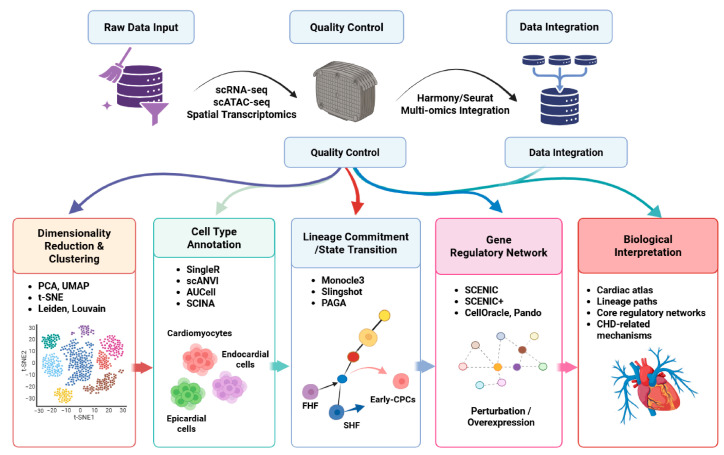
Integrated single-cell multi-omics workflow for cardiac development, from data preprocessing to biological interpretation.

Building on the biological applications outlined above, the analysis of single-cell multi-omics data in cardiac embryonic development has evolved into a biological question-driven framework. As illustrated in Figure 2, this workflow spans from initial data processing to the sophisticated modeling of cell fate regulation. In this section, we discuss how specialized statistical models and computational tools are applied to address specific challenges in cardiac research, such as identifying rare progenitor populations, characterizing evolutionary conservation across species, and reconstructing the complex branching trajectories of heart fields. By integrating these computational strategies, researchers can better link molecular changes to heart morphogenesis, providing deeper insights into the origins of structural defects.

### 4.1. Resolving Cardiac Cell Heterogeneity via Targeted Data Integration and Quality Control

Preprocessing and quality control (QC) are the cornerstones of ensuring that downstream biological insights—such as the identification of rare cardiac progenitor populations—are not artifacts of technical noise. In cardiac embryonic research, the primary challenge lies in the high degree of temporal and spatial heterogeneity [48]. Samples are often collected across broad developmental windows and across different platforms, leading to pronounced batch effects, variable sequencing depths, and ambient RNA contamination from highly transcriptionally active cell populations such as cardiomyocytes [61,62,63].

Early QC methods often relied on rigid, universal thresholds for mitochondrial transcript proportion or detected gene counts, which may not adequately account for dataset-specific or biologically meaningful variation in developing cardiac cells [64]. Modern statistical frameworks provide more nuanced solutions. For instance, SoupX addresses ambient mRNA contamination in droplet-based scRNA-seq data by modeling background RNA and estimating contamination fractions for correction [62]. Likewise, to reduce the risk that transitional states in the developing heart are misclassified, DoubletFinder and Scrublet use artificial doublet simulation and nearest-neighbor-based classification to identify and remove doublets that might otherwise be mistaken for hybrid cell states during lineage specification [65,66].

A major hurdle in constructing a continuous atlas of heart development is the integration of datasets generated across different embryonic stages, platforms, and laboratories [67]. Although the empirical Bayes framework implemented in ComBat provides a robust foundation for correcting additive batch effects, aggressive correction strategies may obscure subtle transcriptomic differences that are biologically meaningful in early cardiac lineage segregation [68]. To better preserve these signals, methods such as Harmony have been widely adopted because they align datasets in a shared embedding while maintaining cell-type-specific structure [69]. In parallel, Seurat’s Canonical Correlation Analysis (CCA)-based and anchor-based integration frameworks have proven effective for integrating datasets across conditions, technologies, species, and even modalities. By identifying shared cellular states across batches, these methods enable projection of data from distinct developmental stages into a unified low-dimensional space, thereby helping distinguish technical variation from genuine developmental trajectories of cardiac precursors [70,71].

### 4.2. Precision Identification of Cardiac Cell Identities and States

Defining cell types and states is a central challenge in single-cell analysis. In the developing heart, this task includes distinguishing subtle populations such as the first and second heart fields, as well as localized progenitor niches within the developing outflow tract [72].

Classical clustering methods, including K-means and hierarchical clustering, combined with Principal Component Analysis (PCA), often struggle with the non-linear, high-dimensional transitions inherent in embryonic heart development [67,73]. These methods are sensitive to initial parameters and frequently fail to resolve the overlapping transcriptomic signatures of cells in the process of lineage commitment, leading to unstable clustering results that may obscure transient intermediate states [74]. Consequently, graph-based clustering algorithms, particularly Louvain and Leiden, have become widely used in cardiac atlas construction. By capturing the complex topological structure of the data, these algorithms are robust to noise and have been extensively used to partition diverse sub-lineages in the developing heart [75]. For instance, they allow researchers to separate closely related endocardial and endothelial subpopulations, though the results remain dependent on the resolution parameters and K-value selection, which must be carefully tuned to avoid over-clustering biologically identical cells [76].

To address the inherent technical noise and dropout-associated uncertainty in early embryonic heart samples, probabilistic generative models have introduced a more rigorous statistical framework. Single-cell Variational Inference (scVI) uses a Variational Autoencoder (VAE)-based Bayesian framework to model the count structure, technical variation, and latent biological signals in scRNA-seq data [77]. In studies of heart development, such latent representations are particularly valuable for cross-sample integration and for resolving major developmental bifurcations, including the divergence of atrial and ventricular lineage. By explicitly accounting for uncertainty in low-abundance transcripts, these models also provide a robust basis for the characterization of rare cardiac cell populations, such as early sinoatrial node-related precursors, although they typically require greater computational resources [78].

Regarding cell type annotation, the challenge in cardiac research is the reliance on a few “canonical” markers, which may not capture the full diversity of the developing organ. Statistical methods like AUCell, which calculates gene set activity scores, and SCINA, based on semi-supervised models, facilitate the preliminary identification of major cardiac compartments [79,80]. Furthermore, as cross-species comparison (e.g., mouse-to-human) is vital for validating cardiac developmental mechanisms, supervised learning tools like SingleR and scANVI have become indispensable. By leveraging high-quality reference datasets from established cardiac atlases, these methods enable the automated and accurate annotation of conserved progenitor states across species, ensuring that specific cell identities—such as epicardium-derived cells (EPDCs)—are consistently identified across different experimental platforms [81,82].

### 4.3. Modeling Cardiac Lineage Trajectories and Fate Decisions

Developmental trajectory inference is a cornerstone of cardiac research, serving to reconstruct the continuous transition pathways of cardiomyocytes, epicardial cells, and endothelial cells. Its primary objective is to transform static single-cell snapshots into a dynamic pseudotime continuum, providing a computational framework to reveal the molecular mechanisms governing cell fate determination [83]. In the complex landscape of the embryonic heart, where progenitors undergo rapid and divergent differentiation, selecting an appropriate mathematical model is crucial for capturing true biological transitions while minimizing technical noise [84].

For relatively linear differentiation processes, such as the maturation of a committed cardiac progenitor into a functional cardiomyocyte, methods based on the Minimum Spanning Tree (MST), represented by Monocle2 (DDRTree), offer an intuitive and smooth representation of the differentiation axis [85,86]. However, the early heart is characterized by highly complex branching events, such as the multipotent differentiation of SHF progenitors into the right ventricle, outflow tract, and atrial components [87]. In these scenarios, MST-based models often struggle to accurately depict simultaneous multi-lineage bifurcations, potentially leading to oversimplified or collapsed trajectory structures [85].

To resolve these complex topologies, graph-theory-based methods like Partition-based Graph Abstraction (PAGA) and Diffusion Maps have become essential. PAGA provides a topology-preserving global connectivity map that is uniquely suited for analyzing the large-scale divergence of myocardial cells into distinct ventricular and atrial lineages [88,89]. By combining global cluster-level connectivity with fine-grained local cell transitions, PAGA can effectively handle the “noisy” connectivity often observed in transitioning cardiac populations. Similarly, probabilistic models such as Slingshot build smooth principal curves that are highly effective for modeling the gradual acquisition of mature cardiac features [90]. While Monocle3, leveraging Leiden clustering, offers enhanced robustness in handling multi-branch scenarios across large-scale cardiac atlases, it remains sensitive to high-dimensional noise, which can lead to “false branches” in the highly heterogeneous early heart environment [91].

Despite these advancements, significant challenges remain in precisely quantifying the uncertainty at critical cardiac branch points, such as the exact moment a progenitor commits to a valvular versus a myocardial fate. The objective definition of “root nodes”—typically representing the nascent mesodermal state—and the mitigation of stochastic high-dimensional noise remain pivotal challenges in ensuring the topological accuracy of heart field lineage reconstructions [83]. These computational uncertainties directly impact our ability to analyze cardiac development mechanisms with high precision. Consequently, the ongoing optimization of trajectory algorithms is not merely a bioinformatic pursuit but a necessity for uncovering the subtle regulatory shifts that, when disrupted, result in complex congenital heart defects.

### 4.4. Deciphering GRNs for Heart Cell Fate Determination

From the “what” of cell maps to the “why” of GRNs, resolving the gene regulatory programs that drive cell fate choices is key to understanding the mechanisms of heart development [92]. In heart development, cell fate choices are governed by complex GRNs where transcription factors (TFs) bind to distal regulatory elements, such as enhancers, to modulate gene expression. By integrating scRNA-seq (measuring expression output) with scATAC-seq (detecting chromatin accessibility), researchers can link open chromatin regions to potential target genes, thereby inferring enhancer–promoter interactions that define the epigenetic landscape of the developing heart [93,94]. A central challenge in cardiac biology is identifying how key TFs, such as GATA4, NKX2-5, and TBX5, coordinate the expression of structural genes during septation or chamber specification [95]. Traditional GRN inference tools like SCENIC predict networks based on co-expression and cis-regulatory motifs [79]. However, to capture the full regulatory logic of the heart, the updated SCENIC+ framework has been developed to incorporate scATAC-seq data, allowing for the inference of enhancer-driven GRNs [96]. This is particularly critical for identifying “shadow enhancers” or redundant regulatory elements that ensure the robustness of heart development [97].

Furthermore, a new generation of tools is moving beyond static network mapping toward dynamic perturbation simulation. Tools like Pando integrate multimodal data to resolve how the spatial environment influences GRN topology. Notably, computational frameworks such as CellOracle empower researchers to perform in silico perturbations, simulating the transcriptomic consequences of transcription factor knock-outs or forced over-expression to predict systemic shifts in the cardiac regulatory landscape [98]. In the context of CHD, such approaches enable researchers to model how disruption of a key TF, such as NKX2-5, may reshape the broader regulatory network and ultimately impair cardiac morphogenesis [99]. By mechanistically linking cis-regulatory elements to downstream gene expression, these integrative GRN models transform static single-cell atlases into predictive landscapes for elucidating both homeostatic cardiogenesis and the pathogenic cascades underlying structural heart defects [100].

## 5. Future Perspectives

Single-cell multi-omics technology provides an unprecedented perspective on cardiac embryonic development, and its value has been widely confirmed. However, as a technology system that is still developing rapidly, it still faces a series of substantial challenges in the breadth and depth of its application. A clear awareness of these bottlenecks and a realistic path to breakthrough are essential for the sustained and healthy development of the field.

### 5.1. The Technical Bottleneck from Detection to High-Resolution Observation

The core of the current technological bottleneck lies in obtaining molecular information within cells more completely, clearly, and conveniently. For instance, scRNA-seq suffers from insufficient capture of low-abundance transcripts (such as those encoding key transcription factors and non-coding RNAs), especially in early heart development research. Increasing sequencing depth can be mitigated, but it is costly and inconsistent with the need for high-throughput, and new library construction technologies with high capture efficiency need to be developed [18]. Current mainstream spatial transcriptomics techniques often capture mixed signals from 2 to 10 cells, which blurs individual cell boundaries and complicates the identification of rare cell populations [101]. For clinical research, this limitation can hinder the precise characterisation of the cardiac conduction system or localised progenitor niches, where even subtle spatial shifts in single-cell signalling are critical for understanding the aetiology of complex congenital heart defects. Although MERFISH has high resolution, it has low throughput and high cost, making it difficult to apply to the analysis of embryo development time series [102]. New methods with high resolution, high reusability, and low cost need to be developed. Technical processes such as CITE-seq and 10x Multiome are complex, and the capture efficiency of different molecules is biased. In addition, it is also a challenge to ensure the accurate alignment of different omics data at the cellular level in the actual experimental operation [103,104]. Emerging technologies, such as high-resolution spatial proteomics and long-read single-cell sequencing, hold promise for addressing some of these limitations in the near future.

### 5.2. Data Analysis from Raw Data to Biological Insights

Extracting reliable and novel biological insights from them is a central challenge for computational methods. Cell annotation relies on marker genes and reference datasets, and the annotation results of new cell populations or transitional cells vary greatly, so it is necessary to establish an authoritative reference map of heart development to promote cross-validation and experimental validation to confirm the identity of key cell types [105]. Trajectory inference algorithms such as Monocle and PAGA are mostly “descriptive”. The trajectory depends on parameters and clustering results, and it is difficult to quantify with certainty due to the lack of branch points. Therefore, it is necessary to develop a probabilistic framework and a trajectory model that can quantify uncertainty [90,106]. GRNs inferred by tools like SCENIC, which are based on co-expression, struggle to distinguish between direct and indirect regulatory interactions [79]. Although CellOracle has made progress, its computational prediction needs to be verified by wet experiments [107].

### 5.3. Translation Barriers from Cell Atlas to Functional Verification

A large number of hypotheses generated by single-cell technology need to be verified by functional experiments, which is the key to transforming data into knowledge and the most time-consuming link at present. Single-cell research readily identifies novel cell types or regulatory factors; however, functionally validating these findings is bottlenecked by the low throughput and long cycles of traditional methods like animal knockouts. There is a pressing need to establish high-throughput parallel validation platforms, such as knockdown screens in zebrafish embryos or perturbation assays in heart organoids [108,109]. It is difficult to obtain human embryo samples, and CHD is mostly caused by minor gene variants superimposed on environmental effects, so the intervention effect of a single target is limited [110]. Short-term goals could include elucidating the functions of CHD risk genes within specific cell types and critical developmental windows, thereby supporting precise genetic counselling.

In conclusion, single-cell multi-omics have pushed the study of heart development to a new stage. The future needs to rely on the deep integration of technology, algorithms, and biological experiments, through quantitative analysis of uncertainty, increasing the throughput of functional validation, interdisciplinary solution of clinical problems, step-by-step across the “description–mechanism” and “atlas–therapy” gap, and deepening the understanding and intervention of CHD.

## Figures and Tables

**Table 1:** Comparison of single-cell multi-omics technologies in cardiac embryonic development.

Technology Type	Core Principle	Key Advantage	Optimal Application Scenario & Logical Thread	Limitation
scRNA-seq	Captures mRNA transcripts in single cells to profile gene expression	Resolves cellular heterogeneity, constructs cell atlases and predicts differentiation trajectories	Stage: All stages Logic: Ideal for defining cell-state transitions and characterising novel cell types	Low detection sensitivity for low-abundance regulators (e.g., rare transcription factors)
scATAC-seq	Detects chromatin accessibility via transposase to identify regulatory regions	Reveals epigenetic landscapes and identifies active enhancers/promoters	Stage: Gastrulation & Early Specification Logic: Best for uncovering upstream drivers (e.g., pioneer factors) before gene activation	Cannot directly link open chromatin regions to target genes without multi-omics integration
Spatial transcriptomics	Maps gene expression while preserving the spatial location of cells	Resolves cell–cell interactions and captures spatial signalling niches	Stage: Heart Tube Formation & Morphogenesis Logic: Essential for resolving spatial coordination and signalling gradients (e.g., BMP/WNT)	Resolution often blurs cell boundaries, with a higher cost for single-cell resolution (e.g., MERFISH)
CITE-seq	Simultaneously profiles transcriptome (mRNA) and proteome (surface proteins)	Provides integrated analysis of mRNA and functional protein expression	Stage: Late Maturation & Functionalization Logic: Best for assessing downstream functional effectors at the protein level	Limited to detectable surface proteins, high experimental cost and library complexity

**Abbreviations:** scRNA-seq, single-cell RNA sequencing; scATAC-seq, single-cell assay for transposase-accessible chromatin sequencing; CITE-seq, Cellular Indexing of Transcriptomes and Epitopes by sequencing.

## Data Availability

No new data were created or analysed in this study. Data sharing does not apply to this article.

## Abbreviations

The following abbreviations are used in this manuscript:

3D	three-dimensional
CCA	Canonical Correlation Analysis
CHD	Congenital Heart Disease
CITE-seq	Cellular Indexing of Transcriptomes and Epitopes by Sequencing
DDRTree	Discriminative Dimensionality Reduction via Learning a Tree
GRN	Gene Regulatory Network
hPSC	Human Pluripotent Stem Cell
hPSC-CM	Human Pluripotent Stem Cell-derived Cardiomyocyte
hvCAS	Human Ventricular Cardiac Anisotropic Sheets
hvCOC	Human Ventricular Cardiac Organoid Chambers
lncRNA	long non-coding RNA
IRX1	Iroquois Homeobox 1
NCBP2	nuclear cap binding protein subunit 2
MERFISH	Multiplexed Error-Robust Fluorescence In Situ Hybridisation
PAGA	Partition-based Graph Abstraction
PCA	Principal Component Analysis
PDZ	Primordium-determining Region
PRC2	polycomb repressive complex 2
SCENIC	Single-Cell Regulatory Network Inference and Clustering
scATAC-seq	Single-cell Assay for Transposase-Accessible Chromatin using sequencing
scBS-seq	Single-Cell Bisulfite Sequencing
scChIP-seq	Single-Cell Chromatin Immunoprecipitation Sequencing
scRNA-seq	Single-Cell RNA Sequencing
scVI	Single-cell Variational Inference
SHF	Second Heart Field
SWI/SNF	Switch/Sucrose Non-Fermentable
TBX5	T-Box Transcription Factor 5
t-SNE	t-distributed Stochastic Neighbor Embedding
UMAP	Uniform Manifold Approximation and Projection
VAE	Variational Autoencoder
VEGF	Vascular Endothelial Growth Factor
PPARα	Peroxisome Proliferator-Activated Receptor alpha
ECM	extracellular matrix
QC	Preprocessing and quality control
TF	transcription factor
